# Randomized clinical trial to determine the effectiveness of CO-oximetry and anti-smoking brief advice in a cohort of kidney transplant patients who smoke

**DOI:** 10.7150/ijms.49401

**Published:** 2020-09-23

**Authors:** Rocio Seijo-Bestilleiro, Teresa Seoane-Pillado, Sonia Pertega-Diaz, Cristina González-Martín, Francisco Valdes-Cañedo, Vanesa Balboa-Barreiro, Constantino Fernandez-Rivera, Angel Alonso-Hernandez, Mercedes Cao-Vilariño, Vicente Gil-Guillen, Mª Teresa Garcia-Rodriguez

**Affiliations:** 1 Clinical Epidemiology and Biostatistics Unit, Instituto de Investigación Biomédica de A Coruña (INIBIC), Complexo Hospitalario Universitario de A Coruña (CHUAC), SERGAS, Universidade da Coruña, A Coruña; 2 Grupo de Investigacion Reumatologia y Salud Pública. Investigación en enfermería y cuidados de la salud. Complejo Hospitalario Universitario A Coruña (CHUAC), SERGAS, As Xubias 84, 15006 A Coruña. Universidade da Coruña (UDC) (A Coruña, España).; 3 Nephrology Department, Instituto de Investigación Biomédica de A Coruña (INIBIC), Complexo Hospitalario Universitario de A Coruña (CHUAC), SERGAS, Universidade da Coruña, A Coruña; 4 Department of Clinical Medicine; University Miguel Hernandez of San Juan de Alicante

**Keywords:** “Smoking cessation”, “Kidney transplantation”, “Controlled clinical trial”, ” Carbon monoxide”, ”Nicotine dependence”.

## Abstract

**Background**: measure the efficacy of exhaled carbon monoxide (CO) measurement plus brief advisory sessions to reduce smoking exposure and smoking behaviour in kidney transplant recipients.

**Methods**: Randomized, controlled, open-label clinical trial at a Spanish hospital.Smoking kidney transplant recipients giving their consent to participate were randomized to control (brief advice, n=63) or intervention group (brief advisory session plus measuring exhaled CO, n=59).

Measurements: Sociodemographic characteristics, cardiovascular risk factors, treatment, rejection episodes, infections, self-reported smoking, drug use, level of dependence and motivation to stop smoking (Fagerström's and Richmond's test) and stage of change (Prochaska and DiClemente's Stages). Efficacy was assessed at 3, 6, 9 and 12 months as: cotinine test, CO levels in exhaled air, nicotine dependence, motivational stages of change, motivation to stop smoking, pattern of tobacco use and smoking cessation rates.

Logistic regression models were computed.

**Results**: At 12 months of follow-up, differences were found in exhaled CO between the intervention and control group(6.1±6.8vs.10.2±9.7ppm;p=0.028). Carboxyhemoglobin levels were lower in the intervention group as well as the positive cotinine test (1.2±1.2%vs.2.0±2.4%;p=0.039),(53.4%vs.74.2%). At 12 months, intervention reduces the probability of a positive urine test by 28%.

**Conclusions**: Co-oximetry is a clinically relevant intervention for reduction of tobacco exposure in kidney transplant recipients.

## Background

Smoking is a major public health issue that leads to avoidable mortality and morbidity. It is associated with cardiovascular disease, cerebrovascular disease, arteriosclerosis, cancer and all-cause mortality 1, 2.

Nicotine acts on cholinergic receptors through sympathetic stimulation. It boosts the release of neurotransmitters such as epinephrine, dopamine, acetylcholine, serotonin, vasopressin, glutamate, nitric oxide, calcitonin and beta endorphin. It is for this reason that nicotine may contribute to an increased risk of cardiovascular episodes caused by temporary surges in arterial pressure, coronary vasoconstriction and damage to the endothelial wall 1, 3.

There is extensive documented evidence of the action of tobacco on renal function as a predictor of chronic kidney disease 4. Furthermore, we found a bibliography that links tobacco with renal function as a predictor of chronic kidney disease^5^.

Literature has documented the connection between smoking and kidney failure among the general public: examples include MRFIT studies^6^, the study by Pinto-Sietsma et al.^7^, PREVEND^8^, the study by Briganti et al.^9^ and the study conducted by Verhave et al.^10^. This connection has also been detected in diabetic patients as a factor that accelerates kidney failure, shortening the time before dialysis is required^11^.

The presence of chronic kidney disease is considered to be independent of the cardiovascular risk marker. Preventing or delaying the appearance of chronic kidney disease is essential, as it is associated with a greater cardiovascular risk (CVR) which increases in line with the degree of kidney failure and albumin excretion rates. This association between the degree of decreased glomerular filtration and a higher CVR is also present in relation to the risk of death and hospitalization^12^.

Clinical evidence also indicates that smoking has serious negative effects on a number of kidney diseases, including diabetic nephropathy, hypertensive nephropathy and primary glomerular diseases, as well as on patients on chronic haemodialysis and in kidney transplanted patients, who are at a greater risk of cardiovascular disease and cancer than the general population^13^. Smoking is therefore a considerable risk factor in this patient group.

Studies have revealed that the prevalence of smoking varies enormously among kidney transplant recipients. Earlier research carried out by our group showed that up to 41.7% of kidney transplanted patients at our centres were smokers at the time of transplant and around 15% continued to smoke following their transplant^14^. Although this figure is not particularly high, the impact of smoking on the probability of cardiovascular episodes in monitoring kidney transplant recipients is clinically relevant. As stated in an earlier article^14^, the number of patients need to treat (NNT) that would have to stop smoking to prevent a cardiovascular event at 5 and 10 year follow up was 7 and 4 respectively.

A systematic review^15^ identified 12 studies during the period between 1968 and 2009 reporting on the effect of smoking on kidney transplant survival. Although the results of some of the studies reviewed were contradictory, in general, smoking was linked to a higher risk of graft loss and death. In turn, other studies have shown that smoking is associated with a higher rate of graft loss and lower survival levels of kidney transplanted patients^16^.

The benefits of giving up smoking have been proved in a wide group of cardiovascular disease patients^17^, ^18^.

Non-pharmacological methods are inexpensive and easy to implement and can also be particularly suitable for transplant patients, who are usually polymedicated. Non-pharmacological interventions to treat tobacco dependence include advice from health professionals, self-help, proactive telephone advisory services, group or individual counselling, material support and social treatment^19^.

All smokers should receive advice on how to stop smoking. Even relatively brief advice has been proved to be effective^20^.

A number of projects are currently underway to determine the effectiveness of co-oximetry and brief advice on giving up smoking^21^, ^22^. Measuring CO levels in the air exhaled by smokers could encourage them to stop smoking or prove a useful tool in monitoring progress in the process of giving up.

Despite the proven impact of smoking on cardiovascular risk and the progression of chronic kidney disease, we have been unable to find any studies that assess the effects of anti-smoking interventions in patients with chronic kidney failure or in kidney transplant patients^23^.

The performance of a co-oximetry, a simple test in which the patient can see a number and a color that indicates the amount of carbon monoxide and exhaled carboxyhemoglobin and with which the patient can see if it has improved compared to previous visits, has proven to be more effective in achieving cessation of smoking 24,25.

The object of this study is to determine the efficacy of measuring exhaled carbon monoxide (CO) by Co-oximetry, combined with brief advice, on giving up smoking at 12 months, in comparison with brief advice only, in smoking kidney transplant recipients at the pre-contemplation and contemplation or preparation stages, in relation to the following outcomes: negative cotinine urine test; carbon monoxide levels in exhaled air (CO) by Co-oximetry; degree of nicotine dependences (Fagerström's test); motivation for change (Prochaska and DiClemente's stages of change); motivation to stop smoking (Richmond's test); self-reported smoking and smoking cessation rates.

The object of this study is to determine the efficacy of measuring exhaled carbon monoxide (CO) by Co-oximetry, combined with brief advice, on giving up smoking, in comparison with brief advice only, in smoking kidney transplant recipients.

## Methods

**Fig 1:**
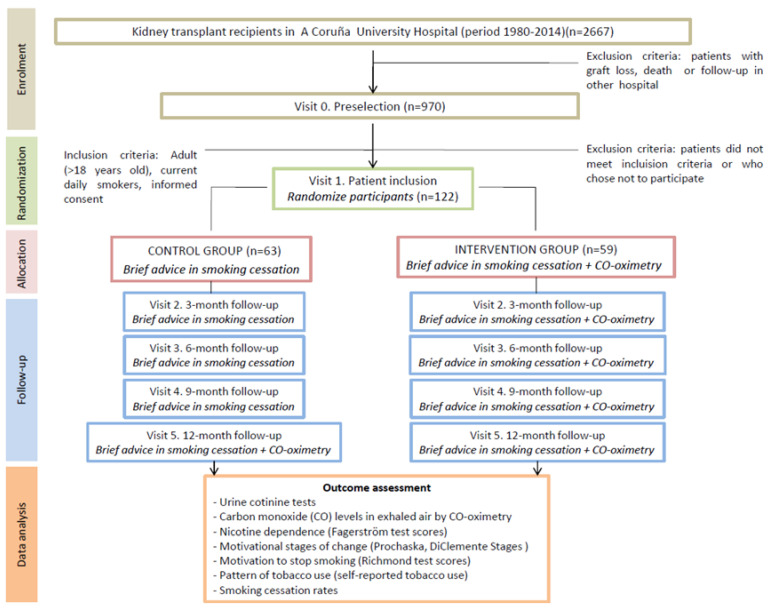
CONSORT flow chart.

The design of this study was previously published 26 in accordance with CONSORT guidelines. Participant flow chart is shown in Fig 1.

Briefly, smoker patients who met eligibility criteria were recruited in the scheduled routine visits to the hospital and informed about the study objectives. After giving informed consent to participate in the study, patients were randomized either to the control or the intervention group according to the following protocol:

a) Control group: brief smoking cessation advice session 27

b) Intervention group: brief smoking cessation advice plus exhaled CO measurement by CO-oximetry

### Trial design

Randomized, controlled and open-label clinical trial with parallel groups set in the Nephrology Department at the Complexo Hospitalario Universitario A Coruña (northwest Spain).

This study was registered after patient recruitment began but before completion of data analysis, as both our institution and the funding agency only required the approval of a Research Ethics Committee to conduct the study. Trial registry can be found at the ISRCTN registry (Identifier: ISRCTN16615772) and with the European Clinical Trials Database (EudraCT number 2015-002009-12).

### Participants

All kidney transplanted patients attending specialized consultations at the Nephrology department during the study period (January 2012- December 2015), who met the inclusion criteria, were eligible to participate in the study.

### Inclusion criteria

- Adult (over 18 years of age) kidney transplant recipients who smoked, with functioning allograft, who underwent primary or repeated renal transplantation from a cadaveric or a living donor in the A Coruña Hospital between 1981 and 2014.
- Current daily smokers. Smoking status was defined according to the WHO classification. Therefore, a patient was considered a current daily smoker if they reported daily smoking during the previous month, irrespective of the number of cigarettes smoked.
- Patients in the pre-contemplation, contemplation or preparation stages, based on Prochaska and DiClemente's transtheoretical model 28.

### Exclusion criteria

- Smokers attempting to stop smoking, patients with terminal illness or mental disability that prevented them from participating.

### Interventions

During the baseline visit (visit 1) patients included in the study control group were given a brief advisory session about giving up smoking. This was firm, concise, personalized (trying to find the most important motivations for each patient) and appropriate to the phase towards cessation they were in. It included information about the negative effects of tobacco on their health and an insight into the principal advantages of giving up smoking^27^.

Patients included in the intervention group received the brief advisory session, as did the patients who belong to the control group, who were also administered Co-oximetry. This exploration determines amount of CO present in the subject's body. The patient took a deep breath and held it for 15 seconds, followed by a slow, long and complete exhalation. After a few seconds the oximeter indicator became stable and marked the exact number of particles per million (ppm) of CO in the subject's exhaled air. Follow-up visits took place at 3 months (visit 2), 6 months (visit 3) and 9 months (visit 4). At each of these moments the anti-smoking advice was repeated in the control group and anti-smoking advice plus Co-oximetry repeated in the intervention group (Table 2). At the 12-month (visit 5) visit, the anti-smoking advice was given to both groups as well as Co-oximetry.

The device used was Bedfont Micro Smokerlyzer Breath Carbon Monoxide Monitor, made in UK. It has been calibrated prior to its use. We do not include it in methods for not advertising it, we understand that there are several in the market.

### Outcomes

The effectiveness of both interventions was assessed at 3, 6, 9 and 12 months after inclusion in the study. The following outcomes were investigated:

Smoking habit cessation, confirmed by a negative urinary cotinine test (intervention was considered effective if the test results were lower than 100 ng/ml), Carbon monoxide (CO) levels in exhaled air measured by Co-oximetry, Change in nicotine dependence (measured according to the Fagerström test^29^), Variation in the motivational stage of change (according to the Prochaska and DiClemente's Stages of Change model 28), Change in motivation to giving up smoking (according to the Richmond test 30), Self-reported abandonment of smoking and a Reduction in the number of cigarettes smoked per day as reported by the patient.

This measure corresponding to the threshold <100ng / ml was used given that it was the one used in the test strips with which the study was carried out.

### Sample size

Meta-analyses have shown that around 5-6 % of smokers stop after the brief advisory session from a physician 27. This study was designed to include n=120 patient (60 per group). This sample size would allow to detect as statistically significant differences in exhaled CO values of 5 ppm of higher (standard deviation +-10 ppm) between groups, as well as a 18% increase in the smoking cessation rate, with a 95% confidence (alfa=0.05) and 80% power (beta=0.20)​.

### Randomisation

Computerized allocation to each of the study groups was conducted in advance. The assignment sequence was generated by a person who was not responsible for determining patient eligibility. By the nature of the interventions neither the researchers nor the patients could be blinding to the assignment.

### Blinding

This was an open clinical trial. Only those assessing outcomes were blinded after assignment to interventions.

### Statistical methods

Comparability of intervention and control groups was checked in terms of the similarity of the distribution of the variables of interest at baseline. The response that patients rate, at different time points in the follow-up, was compared in both arms of the study according to the outcomes studied.

The chi-square test or Fisher's exact test was used to compare proportions. Student's t test was used to compare means between groups with normal distribution data. The Mann-Whitney test was used to compare quantitative variables between groups in case of a non-normal distribution, determined by the Kolmogorov-Smirnov test.

Correlations between quantitative measurements was determined by the Spearman's rho correlation coefficient. Matched-pair data analysis was also computed. Therefore, to evaluate the differences within each group at different time points, McNemar's test and Wilcoxon's signed ranks test were calculated.

Additionally, clinical relevance of intervention was studied by calculating the relative risk (RR), relative risk reduction (RRR), absolute risk reduction (ARR) and patient number needed to treat (NNT) at different times during follow-up. All these measures were presented with their confidence interval at 95 %.

To analyze the association of using Co-oximetry in each of the outcomes, adjusting for potential confounders, multivariate linear and logistic regression models were employed.

Variables with statistical significance p <0.10 in the bivariate analysis were selected for inclusion in the multivariate regression analysis. A modelling strategy of successive step-wise regression.

The degree of agreement between self-reported smoking, Co-oximetry results and the test results of urinary cotinine levels at 3, 6, 9 and 12 months after surgery was considered and assessed by means of the kappa index.

The validity of self-reported smoking by patients regarding the results of the Co-oximetry and urinary cotinine test was studied. Sensitivity, specificity and positive and negative predictive values were determined, together with their 95 % confidence interval.

All analyses were conducted by intention-to-treat. Analyses were conducted using the statistical package Statistical Package for the Social Sciences software, version 19.0 (SPSS, Chicago, IL, USA).

### Ethics

Informed consent of the patients and the approval from the regional Ethics Committee for Clinical Research (Comité Autonómico de Ética da Investigación de Galicia, EC registry code 2011/061) was obtained.

## Results

### Baseline data

The characteristics of the intervention and control groups are shown in Table 1.

This table reveals no significant differences in socio-demographic variables or co-morbidity pre or post-transplant, or in the follow up times between both groups. Specifically, the groups are comparable in the case of the following variables: transplant age; transplant time in years; patient age at the start of the clinical trial and gender. Likewise, no significant differences were observed in pre-transplant co-morbidity between both groups in terms of the pre-transplant dialysis time; total cholesterol level; prevalence of obesity; prevalence of pre-transplant diabetes; left ventricular hypertrophy; pre-transplant cardiovascular episodes and previous malign tumours. No differences were detected post-transplant between the two groups in terms of cold ischemia time; prevalence of obesity and diabetes; left ventricular hypertrophy; post-transplant tumours; post-transplant cardiovascular episodes; appearance of first-time diabetes following transplant; acute rejection episodes and post-transplant tumours. Furthermore, there are no differences in follow-up time after transplant.

Table 2 shows the characteristics of smoker transplanted patients, as they were randomised to the intervention or control groups, in relation to exposure to tobacco at the baseline assessment phase. Tobacco exposure was assessed in accordance with the following factors: self-reported smoking; nicotine dependence; motivation to stop smoking and the smoking habit cessation stage. This table reveals that the groups are comparable in terms of self-reported smoking; nicotine dependence (Fagerström test); motivation to stop smoking (Richmond test) and the smoking cessation stage (Prochaska and DiClemente stages).

### Outcomes and estimation

The assessment of the follow-up results is shown in Table 3, at 3, 6, 9, and 12 months following intervention.

Significant differences were observed at 12 months between the intervention and control groups in the carbon monoxide exhaled by co-oximetry (ppm) (6.1±6.8 vs. 9.7±10.2 ppm; p=0.028), as well as in the carboxyhaemoglobin exhaled by Co-oximetry (% COHb) (1.2±1.2 vs. 2.0±2.4%; p=0.039). The values of positive cotinine urine were also statistically different between the intervention group and the control group at the end of the study (p=0.018): in the case of the intervention group positive prevalence stood at 53.4%, compared with 74.2% in the control group. Statistically significant differences were also observed between the intervention and control groups at various stages of the follow-up in terms of the smoking habit cessation stage based on the Prochaska and DiClemente stages: at 12 months there were more patients at the smoking habit cessation stage in the intervention group than in the control group (46.6% vs 32.3%). The same trend was observed at 6 months. No significant differences were registered between the groups in terms of the following variables: the self-reported smoking (number of cigarettes per day); smoking rates (%), nicotine dependence (Fagerström test) and motivation to stop smoking (Richmond test). Although no differences were observed between the two groups in terms of the self-reported smoking (number of cigarettes per day), this value proved to be slightly higher among the control group than the intervention group throughout the follow-up period and the same trend was observed in the case of nicotine dependence (Fagerström test).

As for the losses in each group, say that at 3 and 6 months there was no loss, both in the control group and in the intervention. At 9 months there was a loss in the intervention group and at 12 months a loss in the control group.

Concordance between self-reported smoking and the positive cotinine urine during follow-up, measured by the Kappa index, is shown in Table 4. This table shows a high degree of concordance between both measurements. A number of other variables were also assessed, including the sensitivity, specificity and predictive values of self-reported smoking in relation to the results obtained from the cotinine in urine test. The results revealed high sensitivity, specificity and predictive value levels. The low levels of negative likelihood ratios indicate that patient negation of exposure to smoking does not rule this out. In contrast, the positive likelihood ratio which registered such high values such as those recorded at 9 months (40.41) indicates that a positive answer practically confirms exposure.

**Figure 2:**
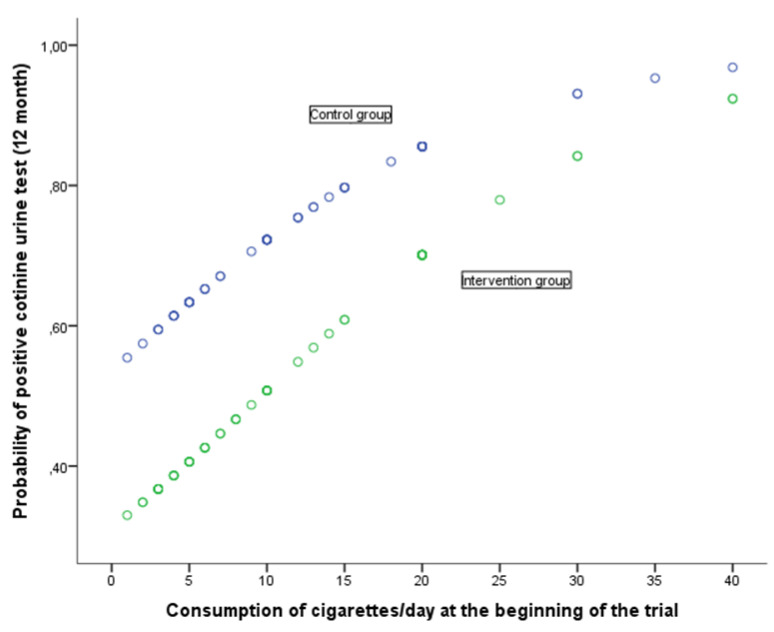
Probability of positive cotinine urine test (12 month) in relation with consumption of cigarettes/day at the beginning of the trial according to group

**Figure 3:**
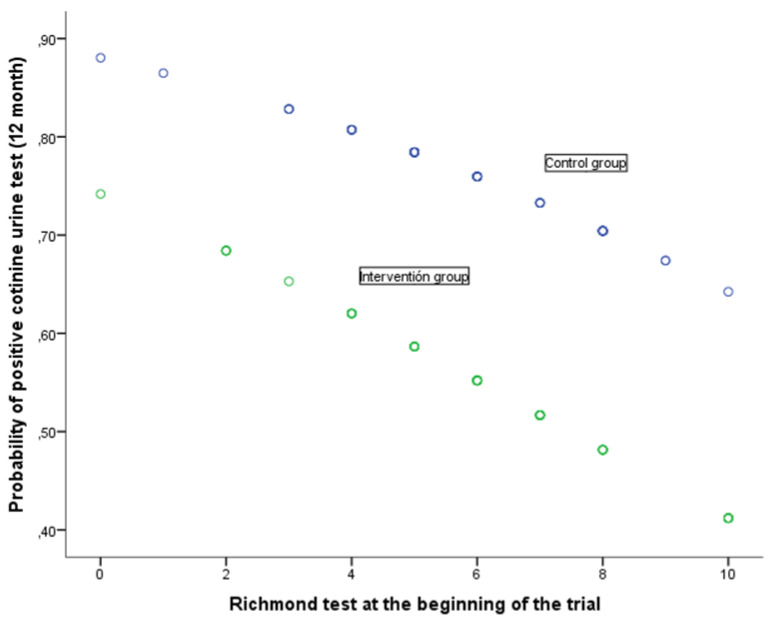
Probability of positive cotinine urine test (12 month) in relation with Richmond test at the beginning of the trial according to group

The relation between the response at 12 months to the cotinine urine test (positive vs. negative) and the various baseline variables together with the assignment group are shown in Table 5. The univariate analysis shows that the response at 12 months is associated with baseline self-reported smoking (cigarettes per day), motivation to stop smoking (Richmond test) and the study group. In the case of the (OR adjusted) multivariate analysis, after considering the variables age at start of trial, self-reported smoking, nicotine dependence (Fagerström test), motivation to stop smoking (Richmond test), gender and assigned group, the following variables were noted as having an independent effect on predicting a positive cotinine urine test: self-reported smoking (cigarettes per day), baseline, motivation to stop smoking measured in accordance with the Richmond test and the group assigned to the study. Forming part of the intervention group implied a significant reduction in the risk of showing a positive cotinine urine test (OR 0.39; 95% IC: 0.17-0.89). In turn, a greater motivation to stop smoking reduced the risk of testing positive (OR 0.83; 95% CI: 0.68-0.99) whilst self-reported smoking (cigarettes per day) increased this risk. The higher the number of cigarettes smoked, the greater the risk of a positive result (OR 1.09; 95%CI: 1.02-1.16). The relationship of baseline self-reported smoking and motivation to stop smoking with the probability of a positive urinary cotinine test at 12 months, in each of the groups of study, is shown in Figs 2 and 3. A higher self-reported smoking and a lower motivation to quit smoking are related to a higher probability of a positive test. On the other hand, patients in the intervention group showed a lower probability of a positive cotinine test.

Cotinine urine test results were used to determine the clinical relevance of the intervention, calculating the associated RR (relative risk), RRR (Relative risk reduction), ARR (absolute risk reduction) and NNT (number needed to treat) at different time points in the follow-up (Table 6). At 12 months, use of Co-oximetry reduce positive test results by 28% in relation to the brief advisory session alone, resulting in 12 patients needed to treat in order for one to quit smoking.

## Discussion

A previous study conducted by our group revealed that patients who continue to smoke following kidney transplant have increased risk of cardiovascular events in comparison with non-smokers. Furthermore, the rate of such episodes also increases in line with exposure time 14. The study revealed clinical relevance regarding the cardiovascular episode rates and giving up smoking.

Of all the possible interventions, motivation to lead a healthy lifestyle and giving up smoking are the simplest and least expensive options that can be applied by means of a brief advisory session and Co-oximetry. Other possible more expensive interventions 31-34 could be drugs, economic incentives, acupuncture, electronic cigarretes, the cost of a coximeter is approximately € 60 and we can use it in all our patients only by changing the mouthpiece.

In this clinical trial, following Co-oximetry, significant reductions were observed in the following variables: carbon monoxide exhaled by Co-oximetry, cotinine urine test and changes in the intervention group and control group in the Prochaska and DiClemente transtheoretical model of change. There is a high degree of concordance between the self-reported smoking and the cotinine urine test. These reductions are consistent with other publications referred to above that prove the effectiveness of this type of intervention 21,22,24,25.

It seems worthy to mention how both interventions, brief advice in smoking cessation and brief advice in smoking cessation + CO-oximetry, remarkably improved some outcomes, such as about a 50% lower number of self-reported cigarettes smoked per day in both groups at the end of the study.

After considering age at the start of the trial, reported smoking, the Fagerström nicotine dependence test, motivation to stop smoking in accordance with the Richmond test, patient sex and the trial group assigned, we observed that the following variables have an independent effect on the prediction of a positive cotinine urine test 12 months: belonging to the study control group, motivation to stop smoking in accordance with the Richmond baseline test and baseline reported smoking. Belonging to the intervention group implies a significant reduction in the risk of positive cotinine urine testing. A greater motivation to stop smoking (by means of the Richmond test) reduces this risk whilst higher self-reported exposure to smoking will increase it.

To the best of our knowledge, this study is the first to prove the effectiveness of co-oximetry in reducing smoking behavior in this patient subgroup. We have been unable to find articles on anti-smoking intervention among patients with kidney failure or kidney transplanted patients^23^. We found articles that recommend regular follow-up of transplant smokers so that they quit smoking 35 In this sense, this research contributes new evidence for the kidney transplanted patient subgroup.

Although it is not the outcome of the trial, it seems worthy to mention how both interventions remarkably improved some outcomes, such as about a 50% lower number of self-reported cigarettes smoked per day in both groups.

### The limitations of this study are discussed below

a) Selection bias: This study can only be applied to this patient subgroup (kidney transplant recipients that smoke) in reducing exposure to smoking. The results may be due to selection bias as it is applicable to a subgroup of transplanted patients that smoke, although this is doubtful as the results are consistent with those obtained with other patient subgroups and at all events, the comparability of the groups at the start of the trial has been proved.

b) In order to minimise information bias, the questionnaires employed were drawn up by skilled professionals.

Questions often arise regarding the validity of the self-reported smoking variable as smokers are generally believed to underestimate the number of cigarettes they actually smoke 36. For the purpose of our study, we considered the validity and concordance of the self-reported results with the biochemical findings. The results indicated a high degree of concordance and validity in this patient subgroup.

In order to avoid classification errors and the tendency towards self-deception shown by smokers, due to the information bias we recommend biochemical measures in order to validate self-reported smoking patterns among patients taking part in the assessment studies^37^. This study uses both the self-reporting smoking variable and biochemical means.

c) For the purpose of minimising any possible confusion regarding other variables, our comparative study addressed not only co-morbidity, but also prior exposure to smoking. Furthermore, and in order to consider the various confusion variables, a logistic regression analysis was carried out that took into account not only the group assigned but also self-reported smoking, nicotine dependence, motivation to stop smoking, age, gender and the transplant time.

## Conclusions

At 12 months the study showed a fall in carbon monoxide and carboxyhaemoglobin levels in the air exhaled by co-oximetry in the intervention group in comparison with the control group. Likewise, the positive cotinine urine test showed significantly lower results in the intervention group than in the control group.

Also at 12 months, the Prochaska and Di Clemente transtheoretical model of change revealed a significant difference between the intervention group and the control group, consisting of a greater prevalence of the smoking cessation stage among the former group in comparison with the latter. The same trend was also observed at 6 months.

No significant differences were observed between the two groups in terms of the self-reported smoking (number of cigarettes per day), smoking rates (%), nicotine dependence (Fagerström test) and motivation to stop smoking (Richmond test). Although no differences were observed in self-reported smoking (number of cigarettes per day) between the two groups, the number was slightly higher in the control group than in the intervention group throughout the entire follow-up period. A similar trend was noted in the nicotine dependence test (Fagerström test). There is a high degree of concordance between the self-reported smoking and the cotinine urine test.

After considering age at the start of the trial, reported smoking, the Fagerström nicotine dependence test, motivation to stop smoking in accordance with the Richmond test, patient sex and the trial group assigned, we observed that the following various have an independent effect on the prediction of a positive cotinine urine test 12 months: belonging to the study control group, motivation to stop smoking in accordance with the Richmond baseline test and baseline reported smoking. Belonging to the intervention group implies a significant reduction in the risk of positive cotinine urine testing. A greater motivation to stop smoking (by means of the Richmond test) reduces this risk whilst higher self-reported exposure to smoking will increase it.

In short, the use of Co-oximetry in kidney transplanted patients that smoke is a clinically relevant intervention for the reduction of exposure to smoking, as indicated by the relative risk reduction, absolute risk reduction and the number of patients needed to treat in order to get one patient to stop smoking.

The effectiveness of Co-oximetry in reducing exposure to smoking is confirmed in this patient subgroup. The intervention is easy to apply, inexpensive and of major importance in the prevention of cardiovascular risk.

## Figures and Tables

**Table 1:** Characteristics of the patients according to the intervention or control group

	Intervention Group (n=59)		Control Group (n=63)		
	**Mean (±SD)**	**n (%)**	**Mean (±SD)**	**n (%)**	**p**
**Age at transplant (years)**	42.8±11.0		43.5±12.4		0.742
**Transplanted time (years)**	5.4±6.2		5.4±5.7		0.718
**Age at start of clinical trial (years)**	48.2±11.0		48.9±11.5		0.745
**Gender**					0.967
Male		41 (69.5)		44 (69.8)	
Female		18 (30.5)		19 (30.2)	
					
**Pre-transplant comorbidity**					
Duration of renal replacement therapy before transplantation (years)	4.0±4.1		4.7±6.3		0.564
Pre-transplant cholesterol (mg/dl)	148.5±38.6		142.0±40.4		0.379
Body mass index (IMC≥30)		9 (16.1)		3 (5.0)	0.068
Pre-transplant diabetes		4 (6.8)		6 (9.5)	0.581
Left ventricular hypertrophy		19 (40.4)		17 (34.0)	0.513
Previous malignancies		1 (1.7)		2 (3.29)	0.519
Cardiovascular events before transplantation		4 (6.8)		5 (7.9)	0.807
					
**Post-transplant comorbidity**					
Cold ischemia time (hours)	18.3±8.4		20.1±7.7		0.268
Obesity (BMI≥30)		11 (19.3)		6 (10.0)	0.154
Post-transplant Diabetes		15 (25.49)		12 (19.0)	0.397
Left ventricular hypertrophy		21 (44.7)		18 (36.0)	0.384
Neoplasms after transplantation		5 (8.5)		3 (4.8)	0.322
Cardiovascular events after transplantation		4 (6.8)		7 (11.1)	0.404
New-onset diabetes mellitus after transplantation		11 (18.6)		6 (9.5)	0.146
Acute rejection episodes		6 (10.2)		11(17.5)	0.245
					
**Follow-up**					
Follow-up time after transplantation (years)	6.8±6.4		6.7±5.7		0.904

**Table 2:** Baseline assessment of tobacco exposure in relation to: self-reported smoking, nicotine dependence, motivation for giving up smoking and motivational stages of change.

	Intervention Group (n=59)	Control Group (n=63)	
	**Mean (±SD)**	**Mean (±SD)**	**p**
**Pattern of tobacco use (cigarettes/day)**			
At what age did you start smoking?	18.0±4.8	17.5±5.3	0.593
How many cigarettes did you smoke before the transplant?	16.6±11.3	16.2±10.4	0.838
How many cigarettes did you smoke at the time of the transplant?	11.7±10.7	10.8±8.5	0.617
What is the consumption of cigarettes at the beginning of the trial?	12±9.1	12.5±8.3	0.772
**Nicotine dependence (Fagerström test)**	1.54±1.8	1.94±2.3	0.545
**Motivation for giving up smoking (Richmond test)**	6.1±2.3	6.1±2.0	0.930
			
	**n (%)**	**n (%)**	
**Positive urine cotinine test**	59 (100)	63 (100)	--
**Fagerström test by categories**			0.153
≤ 4 Low dependence	56 (94.9)	53 (84.1)	
5-6 Moderate dependence	2 (3.4)	6 (9.5)	
≥ 7 High dependence	1 (1.7)	4 (6.3)	
**Motivational stages of change (Prochaska and DiClemente's stages)**			0.950
Pre-contemplation	33 (54.0)	34 (54.0)	
Contemplation	7 (11.9)	7 (11.1)	
Preparation	19 (22.2)	22 (34.9)	
**Richmond test by categories**			0.805
≥7 High motivation	24 (44.4)	23 (42.6)	
5-6 Average Motivation	18 (33.3)	21 (38.9)	
≤4 Low motivation	12 (22.2)	10 (18.5)	

**Table 3:** Outcome measures during the follow-up by group

	3 months			6 months			9 months			12 months		
	IG	CG		IG	CG		IG	CG		IG	CG	
	Mean±SD	Mean±SD	p	Mean±SD	Mean±SD	p	Mean±SD	Mean±SD	p	Mean±SD	Mean±SD	p
**Pattern of tobacco use (Cigarettes/day)**	5.6±6.0	6.2±6.6	0.614	5.2±5.9	5.7±6.5	0.625	4.8±6.1	5.5±6.4	0.532	5.1±6.4	5.6±6.8	0.405
**Pattern of tobacco use (Smoking %)**	39 (66.1)	47(74.6)	0.304	40 (67.8)	46 (73.0)	0.528	34 (58.6)	43 (68.3)	0.271	31 (53.4)	42 (67.7)	0.109
**CO levels in exhaled air by Co-oximetry (ppm)**	6.8±6.8			6.7±7.8			5.8±6.7			6.1±6.8	9.7±10.2	0.028
**%COHb**	1.5±2.0			1.5±2.5			1.1±1.2			1.2±1.2	2.0±2.4	0.039
**Nicotine dependence (Fagerström test)**	0.9±1.2	1.5±2.0	0.338	0.7±1.6	1.3±1.9	0.141	0.9±1.6	1.3±2.0	0.837	1.1±1.6	1.3±2.0	0.976
**Motivation for giving up smoking (Richmond test)**	5.9±1.8	6.1±1.6	0.598	6.3±1.7	6.4±1.8	0.892	6.6±1.6	6.7±1.6	0.869	6.6±1.7	6.1±2.1	0.595
	**n (%)**	**n (%)**	**p**	**n(%)**	**n(%)**	**p**	**n(%)**	**n(%)**	**p**	**n(%)**	**n(%)**	**p**
**Positive cotinine urine test**	42 (71.2)	50 (79.4)	0.294	41(69.5)	48 (76.2)	0.405	35 (60.3)	44 (69.8)	0.273	31(53.4)	46 (74.2)	0.018
**Fagerström test**												
≤ 4 Low dependence	39 (100)	43 (91.5)		38 (95.0)	43 (93.5)		34 (97.1)	39 (90.7)		30 (96.8)	38 (90.5)	
5-6 Moderate dependence	0	2 (4.3)		1 (2.5)	1 (2.2)		0	2 (4.7)		0	2 (4.8)	
≥ 7 High dependence	0	2 (4.3)		1 (2.5)	2 (4.3)		1 (2.9)	2 (4.7)		1 (3.2)	2 (4.8)	
**Motivational stages of change (Prochaska and DiClemente's stages)**			0.518			0.010			0.331			**0.027**
Pre-contemplation	9 (15.3)	11 (17.5)		10 (16.9)	8 (12.7)		13 (22.4)	10 (15.9)		14 (24.1)	9 (14.5)	
Contemplation	16 (27.1)	24 (38.1)		8 (13.6)	25 (39.7)		6 (10.3)	13 (20.6)		4 (6.9)	15 (24.2)	
Preparation	14 (23.7)	12 (19.0)		22 (37.3)	13 (20.6)		16 (27.6)	20 (31.7)		13 (22.4)	18 (29.0)	
Cessation	20 (33.9)	16 (25.4)		19 (32.2)	17 (27.0)		23 (39.7)	20 (31.7)		27 (46.6)	20 (32.3)	
**Richmond test**			0.214			0.694			0.716			0.378
≥7 High motivation	13 (40.6)	19 (46.3)		15 (50.0)	18 (54.5)		8 (47.1)	15 (57.7)		3 (37.5)	5 (55.6)	
5-6 Moderate motivation	11 (34.4)	18 (43.9)		9 (30.0)	11 (33.3)		8 (47.1)	9 (34.6)		5 (62.5)	3 (33.3)	
≤4 Low motivation	8 (25.0)	4 (9.8)		6 (20.0)	4 (12.1)		1 (5.9)	2 (7.7)		0	1 (11.1)	

**Table 4:** Validity, safety and concordance of self-reported smoking in comparison with the determination of urine cotinine test.

	Estimation 95% CI	Estimation 95% CI	Estimation 95% CI	Estimation 95% CI
**Prevalence**	75.41% 66.63-82.55	72.95% 64.02-80.40	65.29% 56.03-73.56	64.17% 54.85-72.57
**Correctly diagnosed**	95.08% 89.15-97.99	97.54% 92.44-99.36	96.69% 91.24-98.94	96.67% 91.17-98.93
**Sensitivity**	93.48% 85.80-97.32	96.63% 89.77-99.13	96.20% 88.55-99.01	94.81% 86.53-98.32
**Specificity**	100% 85.87-99.70	100% 87.02-99.72	97.62% 85.90-99.88	100% 89.79-99.79
**PPV**	100% 94.67-99.89	100% 94.67-99.89	98.70% 91.99-99.93	100% 93.77-99.88
**NPV**	83.33% 66.53-93.04	91.67% 76.41-97.82	93.18% 80.29-98.22	91.49% 78.73.97.24
**Positive likelihood ratio**	--	--	40.41 5.82-280.33	--
**Negative likelihood ratio**	0.07 0.03-0.14	0.03 0.01-0.10	0.04 0.01-0.12	0.05 0.02-0.13
**Kappa index**	0.876	0.939	0.928	0.929

PPV: positive predictive value; NPV: negative predictive value

**Table 5:** Characteristics of the patients according to urine cotinine test at 12 months according to different variables. Univariate and multivariate regression analysis

	Positive urine cotinine test at 12 months	Negative urine cotinine test at 12 months			
	**Mean±SD**	**Mean±SD**	**p**	crude OR* (95%CI)	adjusted OR** (95%CI)
**Transplant age (years)**	42.9±11.2	42.9±12.4	0.996	1.0 (0.97-1.03)	
**Transplanted time (years)**	5.0±5.2	6.1±7.0	0.298	0.97 (0.90-1.03)	
**Age at the start of the clinical trial (years)**	47.9±10.7	49.0±11.9	0.581	0.99 (0.96-1.02)	0.99 (0.96-1.02)
**Pattern of tobacco use (Cigarettes/day)**	14.0±8.9	9.0±7.4	***0.004***	1.09 (1.03-1.15)	***1.09 (1.02-1.16)***
**Nicotine dependence (Fagerström test)**	3.4±1.1	3.1±1.0	0.099	1.37 (0.94-2.00)	1.03 (0.65-1.62)
**Motivation for giving up smoking (Richmond test)**	5.9±2.1	6.9±2.5	***0.026***	0.81 (0.68-0.98)	***0.83 (0.68-0.99)***
					
	**n (%)**	**n (%)**			
**Gender**			0.915		
Female	24(64.9%)	13(35.1%)		1	
Male	53(63.8%)	30(36.2%)		0.92 (0.43-2.12)	0.60 (0.23-1.55)
**Group**			***0.019***		
Control Group	46(74.2%)	16(25.8%)		1	
Intervention Group	31(53.4%)	27(46.6%)		0.40 (0.18-0.86)	***0.39 (0.17-0.89)***

*Crude OR: OR from univariate analysis ** Adjusted OR: OR after logistic regression analysis

**Table 6:** Clinical relevance of the results

	Positive cotinine urine test				
	3 months	6 months	9 months	12 months	12 months 95% CI
**Smoking rates**					
Intervention Group	71.2%	69.5%	60.3%	53.4%	
Control Group	79.4%	76.2%	69.8%	74.2%	
**p value**	0.294	0.405	0.273	**0.018**	
**Clinical relevance**					
RR	0.90	0.91	0.85	0.72	**0.54-0.96**
RRR	0.10	0.09	0.15	0.28	**0.04-0.46**
ARR	0.08	0.07	0.11	0.20	**0.04-0.37**
NNT	12.23	14.93	9.51	4.88	2.68-27.28

RR: relative risk RRR: relative risk reduction ARR: absolute risk reduction NNT: number needed to treat

## Abbreviations

CO: Carbon monoxide
HDL: High-density lipoprotein
LDL: Low-density lipoprotein
NHS: National Health Survey
NNT: Number needed to treat
PPM: Particles per million
ARR: Absolute risk reduction
BMI: body mass index
CI: confidence interval
COHb: carboxyhaemoglobin
CRV: cardiovascular risk
IG: intervention group
CG: control group
RR: Relative risk
RRR: Relative risk reduction
WHO: World Health Organization
NPV: negative predictive value
OR: odds ratio
PPV: positive predictive value
SD: standard deviation
